# Retrospective Evaluation of Patients Treated for Ectopic Pregnancy: Experience of a Tertiary Center

**DOI:** 10.1055/s-0040-1718444

**Published:** 2020-12-21

**Authors:** Özge Kömürcü Karuserci, Seyhun Sucu

**Affiliations:** 1 Obstetrics and Gynecology Department, Faculty of Medicine, Gaziantep University, Gaziantep, Turkey

**Keywords:** ectopic pregnancy, treatment, methotrexate, surgery, expectant management

## Abstract

**Objective** In recent years, there has been an increase in the incidence of ectopic pregnancies; therefore, it is important for tertiary centers to report their approaches and outcomes to expand and improve treatment modalities. The aim of the present study was to evaluate the general characteristics, treatment and outcomes of cases diagnosed with ectopic pregnancy.

**Methods** In total, 432 patients treated for ectopic pregnancy between February 2016 and June 2019 were retrospectively evaluated.

**Results** Overall, 370 patients had tubal pregnancy, 32 had cesarean scar pregnancy, 18 had pregnancy of unknown location, 6 had cervical pregnancy, and 6 had interstitial pregnancy. The most important risk factors were advanced age (> 35 years; prevalence: 31.2%) and smoking (prevalence: 27.1%). Thirty patients who did not have any symptoms of rupture and whose human chorionic gonadotropin (β-hCG) levels were ≤ 200 mIU/ml were followed-up with expectant management, while 316 patients whose β-hCG levels were between 1,500 mIU/ml and 5,000 mIU/ml did not have an intrauterine gestational sac on the transvaginal or abdominal ultrasound, did not demonstrate findings of rupture, and were treated with a systemic multi-dose methotrexate treatment protocol. In total, 24 patients who did not respond to the medical treatment, 20 patients whose β-hCG levels were > 5,000 mIU/ml, 16 patients who had shown symptoms of rupture at the initial presentation, and 6 patients diagnosed with interstitial pregnancy underwent surgery. Patients with cervical and scar pregnancies underwent ultrasound-guided curettage, and no additional treatment was needed.

**Conclusion** The fertility status of the patients, the clinical and laboratory findings, and the levels of β-hCG are the factors that must be considered in planning the appropriate treatment.

## Introduction

Ectopic pregnancy is implantation of the fertilized ovum outside the endometrial cavity. According to various publications 1 2 3 4 its incidence in all pregnancies varies between 1/150 and 1/1,000. 1 In recent years, there has been an increase in the incidence of ectopic pregnancies due to a rise in sexually-transmitted diseases, and accordingly, in pelvic infections. 2 In spite of this increase in its incidence in the last decade, a decrease was achieved in the mortality rates associated with ectopic pregnancy due to the ability to routinely test for β-hCG and the increasingly widespread use of transvaginal ultrasonography. 3 The ability to establish a diagnosis before rupture enables patients to benefit from medical and conservative surgical treatments. 4 The present study aims to investigate the general characteristics, the treatment approaches and the outcomes of cases diagnosed with ectopic pregnancy at our clinic over a three-year period.

## Methods

### Study Design

In total, 432 patients diagnosed with ectopic pregnancy and treated in the Gynecology and Obstetrics Service at Gaziantep University Faculty of Medicine between February 2016 and June 2019 were retrospectively evaluated. The study was approved by the Gaziantep University Ethical Committee under number: 05/2011–16. Age, parity, previous deliveries and spontaneous/interventional abortions, methods of contraception, smoking status, history of previous ectopic pregnancy, tubal sterilization and pelvic surgery, β-hCG levels, ultrasound results, and treatment methods were retrospectively studied.

### Data Collection

Maternal and obstetrical data were collected from the medical record software of the Obstetrics & Gynecology Clinic of the Faculty of Medicine of Gaziantep University.

### Statistical Analysis

Descriptive data were presented as means ± standard deviations, medians, and percentages. The mean data were compared using the non-parametric Mann-Whitney U test. Values of *p* < 0.05 were considered statistically significant. The data were evaluated using the Statistical Package for the Social Sciences (SPSS, IBM Corp., Armonk, NY, US) software, version 22.0.

## Results

Out of 432 patients, 370 had tubal pregnancy, 32 had caesarean scar pregnancy,18 had pregnancy of unknown location, 6 had cervical pregnancy, and 6 had interstitial pregnancy. The mean age of the sample was 32,1 ± 6,3 years (range: 19 to 46 years). Of these patients, 15.3% had a history of 1 or multiple abortions, 13.9% had a history of previous ectopic pregnancy, 13,1% had a history of pelvic inflammatory disease, and 12.5% had a history of previous tubal surgery. In total, 27.1% of the patients smoked, and 31.2% were older than 35 years of age. Regarding the contraception method, 55.5% of the patients did not use any, 16.7% had intrauterine devices, 11.1% used the withdrawal method, 2.8% had tubal ligation, 4.2% used the male condom, 5.5% used oral contraceptives, and 2.8% used the calendar method. A total of 1.4% of the sample were breastfeeding. The results of the endometrial curettage indicated Arias-Stella reaction in 71%, and decidual reaction in 92% of the cases.

**Fig. 1:**
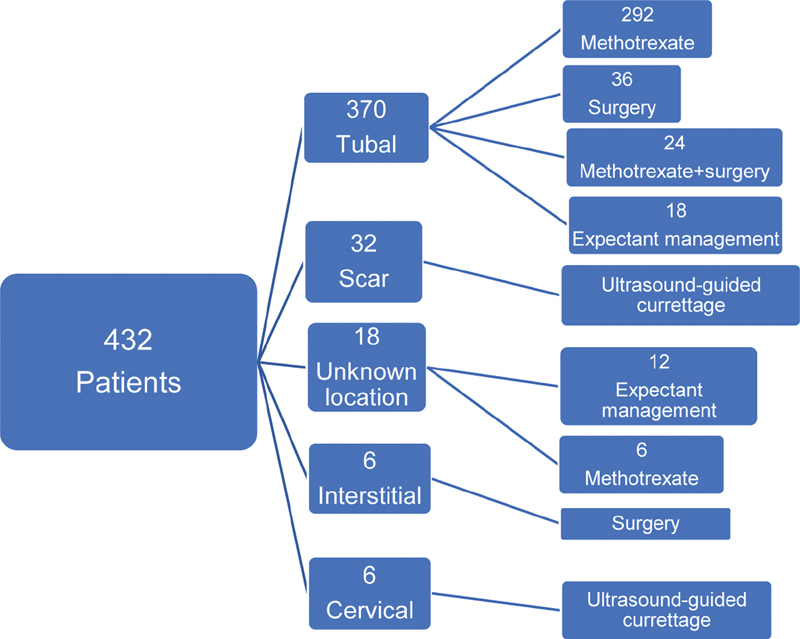
Flowchart of the treatments.

Ectopic pregnancies were diagnosed according to the diagnostic criteria adopted by our clinic. 5 In total, 18 patients who had an adnexial mass on ultrasound and who did not have any symptoms of rupture, and whose human chorionic gonadotropin (β-hCG) level was ≤ 200 mIU/ml were followed-up with expectant management; the β- hCG levels continued to demonstrate a gradual decrease during the follow-up. A total of 316 patients with tubal ectopic pregnancy whose β-hCG levels were between 1,500 and 5,000 mIU/ml, who did not have an intrauterine gestational sac on the transvaginal or abdominal ultrasound, and did not demonstrate findings of rupture were treated with a systemic multi-dose methotrexate treatment protocol. The endometrial curettage was performed as a tool for diagnosis in 67 suspected cases, and the diagnosis was confirmed. The size of the ectopic mass and the fetal cardiac activity did not change the treatment approach for tubal ectopic pregnancies, since it is a relative contraindication. 6 According to this protocol, 1 mg/kg of methotrexate was administered on days 1, 3, 5, and 7, and 0.1 mg/kg of leucovorin was administered on days 2, 4, 6, and 8 intramuscularly, until success was achieved (15% minimum decrease in β-hCG levels based on the tests performed on methotrexate days). 7 The patients were treated surgically if their β-hCG levels on the 9th day did not decrease, or if they showed sypmtoms of rupture during the methotrexate treatment. The success rate of the multi-dose methotrexate treatment was determined as 92.4%. In total, 24 patients underwent surgery after or during the methotrexate treatment. The β-hCG levels of 18 of these patients did not decrease after the medical treatment, and 6 patients showed symptoms of rupture. These 24 patients who did not respond to the medical treatment (all cases of tubal ectopic pregnancies), 20 patients whose β-hCG levels were ≥ 5,000 mIU/ml, 16 patients who had shown symptoms of rupture at the initial presentation, and 6 patients diagnosed with interstitial pregnancy underwent surgery (36 laparoscopic and 30 laparotomic surgeries). Out of the 66 patients who underwent surgery, 48 underwent salpingectomy. A total of 12 patients had tubal abortion, and their abdomens were closed after a lavage of the abdominal cavity, without any additional intervention; 6 interstitial pregnancies underwent wedge resection. Ultrasound-guided curettage was performed in 6 cervical and 32 caesarean scar pregnancies. Following the curettage, these 38 patients showed a progressive fall in their β-hCG levels, and required no additional treatment. In total, 18 patients with pregnancy of unknown location on ultrasound were followed-up with expectant management; 12 of these patients did not need any intervention, and 6 were treated with systemic methotrexate because of an irregular increase in the β-hCG levels. Finally, 68.9% of the patients were submitted to a systemic multi-dose methotrexate treatment, 9.7% underwent surgery, 8.8% were submitted to ultrasound-guided curettage, 6.9% underwent expectant management, and 5.5% were submitted to surgery after the methotrexate treatment. A flowchart of the treatments is shown in Fig. 1 .

The distribution of the cases with regard to the gestational age based on the date of the last menstruation was as follows: weeks 5 to 7 in 52.4% (226); and weeks 8 to 10 in 47.6% (206). At the initial presentation, 16 cases (3.7%) had shown symptoms of rupture. Stable vital signs were observed in 416 cases (91.7%). The delivery and the rates of spontaneous and surgical abortions are summarized in Table 1 ; the number of previous ectopic pregnancies, cesarean sections and pelvic surgeries are summarized in Table 2 ; and the levels of β-hCG among the treatment groups are described in Table 3 .

**Table 1:** Delivery, spontaneous and interventional abortion rates of the patients

		Number of patients	%
Parity	Nullipara	66	15.3
	Primipara	168	38.9
	Multipara	198	45.8
Spontaneous abortion	None	366	84.7
	1	36	8.3
	2	18	4.2
	3	12	2.8
Surgical abortion	None	378	87.5
	1	30	6.9
	2	24	5.5

**Table 2:** Number of previous ectopic pregnancies, cesarean sections, and tubal surgeries

	Number of operations	Number of patients	%
Ectopic pregnancy	0	372	86.1
	1	48	11.1
	2	12	2.8
Cesarean section	0	192	44.4
	1	72	16.7
	2	84	19.4
	3	54	12.5
	4	30	6.9
Tubal surgery	0	378	87.5
	1	42	9.7
	2	12	2.8

**Table 3:** Levels of human chorionic gonadotropin (β-hCG) among the treatment groups

	Number of patients	β-hCG (mIU/ml)	*p* -value
Methotrexate	298	2457 ± 1642	0.123
Surgery	42	5189 ± 4555 ^B^	0.041*
Ultrasound-guided curettage	38	1996 ± 2107	0.813
Expectant management	30	126 ± 78 ^A^	0.021*
Methotrexate + surgery	24	2989 ± 2623	0.442

Note: *Significant at 0.05 level; ^A^ significantly lower than the others; ^B^ significantly higher than the others).

## Discussion

Although the mortality rate in cases of ectopic pregnancy has decreased due to the advances in diagnosis and treatment, its incidence has increased in parallel to the increase in the prevalence of pelvic inflammatory diseases, increasing maternal age, and the increasing use of infertility treatments. 7 The aim of the treatment for ectopic pregnancy has shifted from ensuring survival to preserving fertility, and the conservative surgical techniques were developed to maintain fertility. The treatment options for ectopic pregnancies include expectant management, methotrexate treatment, ultrasound-guided currettage and surgery. The treatment modality should be selected based on the overall condition of the patient, the laboratory findings, and the fertility status. 8 In the present study, we evaluated the approaches we adopted in the treatment of ectopic pregnancy, and discussed our treatment spectrum along with the literature.

In the present study, ectopic pregnancies were most common in the no-contraception group (55.5% ∣ *n* = 240), while intrauterine devices were used by 36 (16.7%) patients. A history of tubal surgery was present in 18 patients (8.3%), and a history of previous ectopic pregnancy, in 30 patients (13.9%). The most important risk factors were advanced age (> 35) and smoking (respectively; 135 (31.2%), 117 (27.1%)). The generally known risk factors for ectopic pregnancy were encountered at moderate rates among the patients included in the present study. 9 10

Studies about expectant management have reported success rates varying between 54% and 92.3%; however, expectant management has the highest success rates in patient groups with low β-hCG levels (≤ 200 mIU/ml). 11 12 In total, 30 patients followed-up with expectant management had significantly lower β-hCG levels compared with the other groups, and our success rate was of 100%. Thus, selecting appropriate patients in the determination of the treatment modality can significantly increase the success rate.

Methotrexate is a folic-acid antagonist, and it inhibits the production of tetrahydrofolate, which is required for the synthesis of DNA, RNA, and ATP. The treatment with methotrexate can be administered locally or systemically. Previous studies determined lower success rates with a single-dose protocol than with a multi-dose protocol, particularly when the β-hCG levels are high. The multi-dose methotrexate treatment appears to be as effective as laparoscopic salpingostomy in the treatment of ectopic pregnancies (the comparison of multi-dose methotrexate with surgery: 82% to 71%; relative risk (RR): 1.15%; 95% confidence interval (95%CI): 0.93–1.43 respectively). 12 A systematic review of randomized studies 13 revealed that single-dose methotrexate (50 mg/m ^2^ , or 1 mg/kg) had a lower success rate than the multi-dose treatment (the comparison of single dose-multidose methotrexate in four studies 14 : 71% to 88%; RR: 0.82%; 95%CI: 0.72–0.94 respectively). Meanwhile, administering an additional dose when a single-dose fails was found to have an effectiveness comparable to that of salpingostomy and the multi-dose treatment (RR: 1.01; 95%CI: 0.92–1.12). 13 14 We have been administering the multi-dose treatment regimen for years, with high success rates; and although the systemic treatment was reported to have side effects such as pneumonia, stomatitis, and alopecia, our patients did not show any side effects, which can be explained by the controlled use of the medication at appropriate doses and on appropriate patients.

The serum β-hCG levels are closely related to the success rates obtained with the medical treatment. A previous study 15 revealed that the initial β-hCG level was the most useful prognostic data to predict the success of the methotrexate treatment. In another study, 16 methotrexate was found to have a success rate of 94% when the initial β-hCG level was lower than 10,000 mIU/ml, and a success rate of 75% when it was higher than 10,000 mIU/ml. More recent publications have shown that a cut-off level between 2,000 mIU/ml 5,000 mIU/ml is more appropriate for high success rates. Similarly, in the present study, we determined higher success rates with an initial β-hCG level below 5,000 mIU/ml.

In the management of cervical and cesarean scar pregnancies, aspiration curettage can be combined with cervical tamponade using a Foley catheter for hemostasis. Besides these procedures, local prostaglandin can be used following the curettage. In case of uncontrollable bleeding, the ligation of the cervical branches of the uterine artery and the ligation of the bilateral hypogastric arteries can be considered. As a last resort, hysterectomy can be performed. 17 In the present study, 6 patients with cervical pregnancies and 32 patients with cesarean scar pregnancies were treated with ultrasound-guided curettage, without any additional interventions. The β-hCG levels of the patients gradually decreased to zero after the curretage. Based on this result, one can state that a successful ultrasound-guided curettage procedure is quite effective as a standalone treatment in patients diagnosed with scar and cervical pregnancies.

Endometrial sampling in the diagnosis of ectopic pregnancy is still controversial. 18 The presence of decidual and typical Arias-Stella reactions in endometrial curettage material without fetal tissue and placental components is an important finding for ectopic pregnancy. Although Arias-Stella reactions were reported to be present at a rate of 40% to 70% in ectopic pregnancies, the presence of a decidual reaction without an Arias-Stella reaction was also reported to corroborate the diagnosis. 19 In line with the literature, the Arias-Stella reaction was determined at a rate of 71% in the cases who underwent endometrial curettage in the present study, and the decidual reaction was determined in 92% of the patients. We must remember that all phases of the endometrium can be found in an ectopic pregnancy, and none are pathognomonic. 20 Nevertheless, examining an endometrial sample can be helpful in patients with unclear differential diagnosis.

The gold standard in the surgical treatment of ectopic pregnancy is laparoscopic surgery, because it is associated with shorter operations and hospitalization, lower blood loss and lower need for analgesics, and a lower total cost. Intra-abdominal adhesion is also less frequent after laparoscopy compared with laparotomy. 21 Meanwhile, laparoscopy may not be the primary choice for patients who are hemodynamically unstable and patients with severe intra-abdominal bleeding. Surgical procedures have a greater impact on future fertility compared with the medical treatment, and the probability of natural conception significantly decreases, particularly after a salpingectomy. On the other hand, the treatment with salpingostomy is typically not preferred by clinicians because of persistent trophoblastic activity and the risk of tubal bleeding. 22 Among the ectopic pregnancies treated at our clinic, those with hemodynamic instability, β-hCG levels higher than 5,000 mIU/ml, and those not responding to the methotrexate treatment were treated with surgical methods. Of such patients, 60% underwent laparoscopy, and 40% underwent laparotomy. Of the 30 patients who underwent surgery, 24 underwent salpingectomy, while 6 had tubal abortions, and the abdominal cavity was irrigated and lavaged without any additional treatment.

## Conclusion

In conclusion, the early and correct diagnosis of ectopic pregnancy is important to prevent mortality and morbidity. It must be taken into consideration that conventional risk factors are not always present, and patients in low-risk groups should be examined thoroughly. In the treatment of ectopic pregnancy, methotrexate therapy, surgical intervention, and expectant approaches have high success rates without complications when administered to the correct patients. Determining the most appropriate treatment approach is of vital importance. The fertility of the patients, the clinical and laboratory findings, and the serum β-hCG levels are factors that must be considered in planning an appropriate treatment.

## References

[1] Demirdag E Guler I Abay S Oguz Y Erdem M Erdem A The impact of expectant management, systemic methotrexate and surgery on subsequent pregnancy outcomes in tubal ectopic pregnancy Ir J Med Sci 2017 186 02 387 392 10.1007/s11845-016-1419-5 26895299

[2] Tsevat D G Wiesenfeld H C Parks C Peipert J F Sexually transmitted diseases and infertility Am J Obstet Gynecol 2017 216 01 1 9 10.1016/j.ajog.2016.08.008 28007229 PMC5193130

[3] Gaskins A J Missmer S A Rich-Edwards J W Williams P L Souter I Chavarro J E Demographic, lifestyle, and reproductive risk factors for ectopic pregnancy Fertil Steril 2018 110 07 1328 1337 10.1016/j.fertnstert.2018.08.022 30503132 PMC6309991

[4] Jauniaux E Jurkovic D Ectopic pregnancy: 130 years of medical diagnostic challenges BJOG 2018 125 13 1672 10.1111/1471-0528.15454 30426656

[5] Tulandi T Ectopic pregnancy: choosing a treatment [Internet] 2020[cited 2010 Feb 10]. Available from:https://www.uptodate.com/contents/ectopic-pregnancy-choosing-a-treatment

[6] Paull C Robson S J Hospital admission and surgical approach to tubal ectopic pregnancy in Australia 2000 to 2014: A population-based study Aust N Z J Obstet Gynaecol 2018 58 02 234 238 10.1111/ajo.12727 29023642

[7] Lipscomb G H Medical therapy for ectopic pregnancy Semin Reprod Med 2007 25 02 93 98 10.1055/s-2007-970048 17377896

[8] Hsu J Y Chen L Gumer A R Tergas A I Hou J Y Burke W M Disparities in the management of ectopic pregnancy Am J Obstet Gynecol 2017 217 01 490 4.9E11 28288792 10.1016/j.ajog.2017.03.001 PMC5484775

[9] Mashiach R Kislev I Gilboa D Mazaki-Tovi S Seidman D S Goldenberg M Bouaziz J Significant increase in serum hCG levels following methotrexate therapy is associated with lower treatment success rates in ectopic pregnancy patients Eur J Obstet Gynecol Reprod Biol 2018 231 188 191 10.1016/j.ejogrb.2018.10.046 30396108

[10] Craig L B Khan S Expectant management of ectopic pregnancy Clin Obstet Gynecol 2012 55 02 461 470 10.1097/GRF.0b013e3182510aba 22510629

[11] Moawad N Baker S Hergert S Shuster J Robinson M Trends in the surgical management of ectopic pregnancy with the addition of MIS faculty J Minim Invasive Gynecol 2017 24(7, Supplement)S81 10.1016/j.jmig.2017.08.216

[12] Mavrelos D Memtsa M Helmy S Derdelis G Jauniaux E Jurkovic D β-hCG resolution times during expectant management of tubal ectopic pregnancies BMC Womens Health 2015 15 43 10.1186/s12905-015-0200-7 25994203 PMC4443555

[13] Mol F Mol B W Ankum W M van der Veen F Hajenius P J Current evidence on surgery, systemic methotrexate and expectant management in the treatment of tubal ectopic pregnancy: a systematic review and meta-analysis Hum Reprod Update 2008 14 04 309 319 10.1093/humupd/dmn012 18522946

[14] Nieuwkerk P T Hajenius P J Ankum W M Van der Veen F Wijker W Bossuyt P M Systemic methotrexate therapy versus laparoscopic salpingostomy in patients with tubal pregnancy. Part I. Impact on patients' health-related quality of life Fertil Steril 1998 70 03 511 517 10.1016/s0015-0282(98)00212-x 9757881

[15] Lipscomb G H Givens V A Meyer N L Bran D Previous ectopic pregnancy as a predictor of failure of systemic methotrexate therapy Fertil Steril 2004 81 05 1221 1224 10.1016/j.fertnstert.2003.09.070 15136080

[16] Alur-Gupta S Cooney L G Senapati S Sammel M D Barnhart K T Two-dose versus single-dose methotrexate for treatment of ectopic pregnancy: a meta-analysis Am J Obstet Gynecol 2019 221 02 95 10800 10.1016/j.ajog.2019.01.002 30629908 PMC6612469

[17] Guzowski G Sieroszewski P Invasive ultrasound in the management of cervical ectopic pregnancy Eur J Obstet Gynecol Reprod Biol 2014 172 7 9 10.1016/j.ejogrb.2013.10.016 24287286

[18] Brady P C New evidence to guide ectopic pregnancy diagnosis and management Obstet Gynecol Surv 2017 72 10 618 625 10.1097/OGX.0000000000000492 29059454

[19] Bestel M Karaaslan O Bestel A Salman S Therapeutic curettage on follow up human chorionic gonadotropin levels in ectopic pregnancy East J Med 2019 24 02 200 203 10.5505/ejm.2019.26818

[20] de Haan J Vandecaveye V Han S N Van de Vijver K K Amant F Difficulties with diagnosis of malignancies in pregnancy Best Pract Res Clin Obstet Gynaecol 2016 33 19 32 10.1016/j.bpobgyn.2015.10.005 26586541

[21] Ades A Parghi S Laparoscopic resection of cesarean scar ectopic pregnancy J Minim Invasive Gynecol 2017 24 04 533 535 10.1016/j.jmig.2016.11.006 27867050

[22] Laganà A S Vitale S G De Dominici R Padula F Rapisarda A MC Biondi A Fertility outcome after laparoscopic salpingostomy or salpingectomy for tubal ectopic pregnancy A 12-years retrospective cohort study Ann Ital Chir 2016 87 461 465 27480601

